# Biomechanical Analysis Using FEA and Experiments of Metal Plate and Bone Strut Repair of a Femur Midshaft Segmental Defect

**DOI:** 10.1155/2018/4650308

**Published:** 2018-10-18

**Authors:** Jason Coquim, Joseph Clemenzi, Mohsen Salahi, Abdurahman Sherif, Pouria Tavakkoli Avval, Suraj Shah, Emil H. Schemitsch, Z. Shaghayegh Bagheri, Habiba Bougherara, Radovan Zdero

**Affiliations:** ^1^Department of Mechanical and Industrial Engineering, Ryerson University, Toronto, ON, Canada; ^2^School of Engineering, Technology, and Applied Science, Centennial College, Toronto, ON, Canada; ^3^Office of Research Administration, Ryerson University, Toronto, ON, Canada; ^4^Orthopaedic Biomechanics Lab, Victoria Hospital, London, ON, Canada; ^5^Department of Surgery, Western University, London, ON, Canada; ^6^Department of Automotive, Mechanical, and Manufacturing Engineering, University of Ontario Institute of Technology (UOIT), North Oshawa, ON, Canada; ^7^Department of Mechanical and Materials Engineering, Western University, London, ON, Canada

## Abstract

This investigation assessed the biomechanical performance of the metal plate and bone strut technique for fixing recalcitrant nonunions of femur midshaft segmental defects, which has not been systematically done before. A finite element (FE) model was developed and then validated by experiments with the femur in 15 deg of adduction at a subclinical hip force of 1 kN. Then, FE analysis was done with the femur in 15 deg of adduction at a hip force of 3 kN representing about 4 x body weight for a 75 kg person to examine clinically relevant cases, such as an intact femur plus 8 different combinations of a lateral metal plate of fixed length, a medial bone strut of varying length, and varying numbers and locations of screws to secure the plate and strut around a midshaft defect. Using the traditional “high stiffness” femur-implant construct criterion, the repair technique using both a lateral plate and a medial strut fixed with the maximum possible number of screws would be the most desirable since it had the highest stiffness (1948 N/mm); moreover, this produced a peak femur cortical Von Mises stress (92 MPa) which was below the ultimate tensile strength of cortical bone. Conversely, using the more modern “low stiffness” femur-implant construct criterion, the repair technique using only a lateral plate but no medial strut provided the lowest stiffness (606 N/mm), which could potentially permit more in-line interfragmentary motion (i.e., perpendicular to the fracture gap, but in the direction of the femur shaft long axis) to enhance callus formation for secondary-type fracture healing; however, this also generated a peak femur cortical Von Mises stress (171 MPa) which was above the ultimate tensile strength of cortical bone.

## 1. Introduction

Surgical repair of recalcitrant nonunions of long bone shaft fractures can be clinically challenging, since they are often accompanied by a large segmental bone defect [1]. Nonunion of long bone shaft fractures can be caused by comminution of the fracture, long bone defects, infection, inferior vascularity of soft tissue and fracture fragments, and poor immobilization [2]. Stable fixation can be difficult due to the influence of disuse osteopenia as patients avoid using the affected limb, age-related osteoporosis particularly for women, and empty screw holes from prior surgery which attempted to treat the original fracture [1]. The result is an unstable long bone with poor bone quality surrounding a nonunited atrophic segmental defect.

Surgically, numerous strategies have been used when bone quality is poor for long bone nonunions and segmental defects for upper and lower extremities. Ring et al. achieved healing in 91% [3] and 100% [4] of problematic osteoporotic humerus shaft nonunions using longer plates (but also blade-type, intramedullary-type, and locking compression-type plates), as well as intramedullary fibular allografts, medial struts, Schuli locking nuts, and cement. Dao et al. obtained healing in 7 months for humerus shaft nonunion repaired with a plate plus an intramedullary fibular allograft augmented with demineralized bone matrix [5]. Crosby et al. treated 12 patients with humerus nonunion in the identical fashion and obtained healing in 10 patients [6]. Wright et al. reported that 8 of 9 patients had union by using a plate plus an intramedullary fibula (allo or autograft) augmented with Iliac crest bone graft [7]. Hornicek et al. treated 10 humerus nonunions with a plate plus a strut and achieved union in 3-6 months [8]. Van Houwelingen and McKee utilized a lateral plate plus a medial allograft strut, along with Iliac crest bone graft or bone morphometric protein, in 6 humeral nonunions which all progressed to union [9]. Wang and Weng described 13 cases of distal femur nonunions repaired with a plate or a nail plus a frozen cortical allograft strut, along with Iliac crest bone graft [10]. Of these, 8 patients had osteoporosis, and 11 patients had femoral defects greater than 20 mm at the site of nonunion. However, full union was obtained in all patients after 5 months. Crowley et al. reviewed prior reports from 1975 to 2004 and discovered that nails, plates, and external fixators are the main option for femoral shaft fracture nonunions with defects up to 50 mm [11].

Biomechanically, however, there is little evidence as to which fixation method is superior for this injury. To potentially augment stabilization using nails and plates for this injury in animal models, various materials have been assessed, such as porous titanium scaffolds, biodegradable polymer-based composite scaffolds, bone autografts, and bone allografts that fill and/or bridge the bone defect to promote union [12–16]. However, only Talbot et al. biomechanically compared multiple reconstruction implants that are commercially available and commonly used clinically for human long bone segmental defects [1]. Axial, torsional, coronal bending, and sagittal bending tests showed that a nonlocking plate plus a medial bone strut had the highest stiffnesses of about 1.3-7.3 times versus other methods, as well as having twice the axial failure force compared to a lateral locking plate alone. Although it provided the highest stiffness, this method was not assessed for the effect of various bone strut lengths, numbers of screws, or locations of screws. This omission is important since strut insertion requires additional soft tissue stripping through a medial approach, which can increase operating time, blood loss, patient discomfort, muscle elevation, and/or bone devascularisation [4]. Moreover, their use of all available cortical screw holes spanning the entire length of the metal plate may be unnecessary for adequate mechanical stability for a femoral midshaft fracture, thus potentially reducing the number of screw insertions and the accompanying bone loss [17].

Therefore, this is the first study to systematically evaluate the biomechanical performance of the metal plate plus bone strut technique for repairing recalcitrant nonunions of a femur midshaft segmental defect versus an intact femur by assessing the effect of strut length and the number and location of screws.

## 2. Methods

### 2.1. General Strategy

FE models were developed to replicate the geometry, material properties, and force application conditions of an intact femur during the single-leg stance phase of walking, as well as after creating a midshaft segmental defect repaired using 8 different configurations involving a metal plate, a bone strut, and bicortical screws. FE models with femurs in 15 deg adduction were analyzed at a subclinical 1 kN axial force for validation against surface strain gage tests (as well as against surface strain data from prior similar studies by the current senior authors) and then also at a clinical-type 3 kN axial force to simulate 4 x body weight during the single-leg stance phase of walking; this generated global axial stiffnesses and local surface Von Mises stresses. Note that throughout this report, the term “axial” means that force was actually applied vertically to the top of the femoral head while femur shafts were oriented in adduction. Finally, the effect of bone strut length, number of screws, and location of screws for this injury were discussed based on both a traditional “high stiffness” femur-implant construct criterion (which could enhance construct stability immediately after surgery) and the more modern “low stiffness” femur-implant construct criterion (which could potentially permit some in-line interfragmentary motion perpendicular to the fracture line, but in the direction of the femur shaft long axis, that is needed for enhanced callus formation during secondary-type fracture healing).

### 2.2. Computational Analysis

#### 2.2.1. Clinical Cases Analyzed

**Figure 1:**
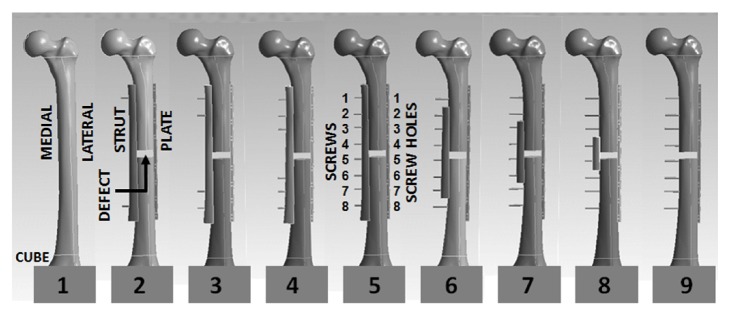
FE CAD model of the artificial femur showing Cases 1 to 9. Geometries, relative positions, and material properties replicated the experiments exactly. Anterior views are shown.

The clinical cases considered were an intact long bone control (i.e., Case 1), several well-known surgical repair methods (i.e., Cases 5, 6, and 9 [1, 8, 9]), and a series of new concepts not previously examined surgically or biomechanically (i.e., Case 2, 3, 4, 7, and 8) for treating long bone segmental defects; all of these cases were replicated by later experiments (Figure 1). Specifically, Case 1 was the intact femur control, which had a 16 mm diameter hollow intramedullary canal and a full length of 485 mm which was inserted into a cement potting cube to a 75 mm depth. Case 2 was the intact femur with a 12 mm defect located at midshaft, i.e., about 183 mm above the top of the cement potting cube. Laterally, the femur was repaired with an 8 hole, 246 mm long, stainless steel, nonlocking plate (Zimmer, Warsaw, IN, USA), whereas medially the femur was fixed with a 246 mm long bone strut created using another identical femur shaft cut along its centerline. Bicortical bone screws made from medical grade stainless steel having a 4.5 mm diameter and a 50 mm length (Model 214.050, Synthes, Paoli, MA, USA) were inserted into screw holes 1/8 through the plate, femur, and strut. Cases 3, 4, and 5, respectively, had additional screws inserted into screws holes 2/7, 3/6, and 4/5. Cases 6, 7, and 8, respectively, had the medial strut symmetrically shortened by cutting it between screw positions 1/2 and 7/8, 2/3 and 6/7, and 3/4 and 5/6, such that Case 9 had no strut at all. Other cases of potential clinical relevance, i.e., alternating the screw insertion sites in mirror-like fashion on either side of the defect, were not considered since their biomechanical performance was likely to fall within the extremes represented by Cases 2, 5, and 9.

#### 2.2.2. CAD Models

For Case 1, the CAD model of an artificial femur (Model 3406, Sawbones, Vashon, WA, USA) was used, having been developed, validated, and employed previously by several present authors and others [17–22]. CT scanning was done on the artificial femur lengthwise every 0.5 mm. Next, CT scans were stocked in DICOM format, imported into Mimics Medical Imaging Software (The Materialise, Group, Leuven, Belgium) to create a 3D model, and thereafter exported into the SolidWorks CAD program (Solid-Works Corp., Dassault Systemes, Concord, MA, USA). The CAD model's surfaces replicated cortical as well as cancellous material, but it did not have an intramedullary canal. The canal was made by removing bone with the “cut” operation from SolidWorks. For Cases 2 to 9, the 12 mm bone gap at the femur's midshaft and the medial strut were made by using the “cut” operation in SolidWorks software.

The femur model's geometry and material properties were identical to those employed in prior works [17–22], except that current properties of cancellous material were changed as detailed below. In addition, tetrahedral elements of the same size and shape were employed to mesh the femur. Regarding mesh refinement and convergence analysis, the model's mesh sensitivity was evaluated with Workbench's “relevance” function. This denotes the minimum (coarse mesh of 0% relevance) to maximum (fine mesh of 100% relevance) number of elements for discretizing the femur. Initial calculations demonstrated that femur models with 80% relevance had stresses and strains less than 1% different from femur models with 95% relevance. Thus, a relevance of 90% was employed throughout.

The CAD program SolidWorks 2010 (SolidWorks Corp., Dassault Systemes, Concord, MA, USA) was also utilized to generate models of the metal plate, metal screws, and cement potting block. Models of these components were based on Vernier caliper measurements of their geometries in the experimental phase, as done before [17–21]. CAD data were saved in SolidWorks with Parasolid file format (*∗*x_t), which ANSYS software easily recognized.

#### 2.2.3. Assembly of Components

Femur-plate, femur-strut, femur-screw, plate-screw, and strut-screw structures were made in SolidWorks from individual models of the femur, strut, plate, and screws. These items were then exported to ANSYS Workbench 12.0 for FEA. The WorkBench “simulation” module automatically generated contact between surfaces that were assembled. CONTA174 in ANSYS is a 3D 8-node surface-to-surface contact element that was used. This contact element was located on a deformable surface of a 3D solid element that contacted and slid on a target surface, i.e., TARGE170 in this study. CONTA174 had 3 degrees-of-freedom at each node; that is, there were translations in the nodal x, y, and z directions. Its geometric characteristics were the same as the face of the solid element face to which it was connected. Contact happened as the element surface pierced the corresponding target element called TARGE170. Also, CONTA174 and TARGE170 had the same real constants. A fully bonded condition was applied to all contact elements.

#### 2.2.4. FEA Meshing and Material Properties

ANSYS Workbench 12.0 software generated meshes for the femur, plate, strut, and screws. From the mesh relevance (i.e., refinement and convergence) assessment described above, the number of nodes (range, 122,255 to 553,009) and elements (range, 72,498 to 344,347) was recorded. A 10-node quadratic tetrahedron body element was used for cortical and cancellous bone as well as all metal parts, while a 20-node quadratic hexahedron body element was used for the axial loading platen. Quadratic triangular contact elements were used for cortical and cancellous bone as well as all metal parts, while quadratic quadrilateral contact elements were used for the loading platen. The artificial femur and strut were linear, elastic, and isotropic with properties for cortical bone (E = 16.7 GPa, *ν* = 0.3) and cancellous bone (E = 0.155 GPa, *ν* = 0.3) [17–23]. The plate and screws were both modeled as medical grade stainless steel (E = 210 GPa, *ν* = 0.3) [17–19].

#### 2.2.5. FEA Boundary Conditions

Assumptions that were similar to those below have been used previously in computational models of femur fracture fixation [17–19, 24]. All femur-plate and femur-strut interfaces were set to “no separation” while allowing some slipping with friction factor = 0.1, but all plate-screw, femur-screw, and strut-screw interfaces were “bonded”. This simulated complete bone on-growth and sufficient plate compression at the various interfaces. Also, bonded contact mimicked full interdigitation of cortical bone into the screw threads, although threads were not modeled. This replicated the long-term clinical scenario whereby bone remodels itself around neighboring bodies. The contact zone between the axial loading platen and the femur's head was estimated as nonslip bonded contact, which simulated later experiments whereby the femoral head was not allowed to move horizontally. Cement potting of the femur's distal condyles as performed in the experiments was approximated as a constraint of the femur's distal 75 mm in the Simulation utility. This was done by restraining the movement of the femur's distal end by assigning movement restrictions for the femur's distal faces. An axial (i.e., vertical) force was applied on the loading platen's face with movement restricted along the x- and y-axes, but not the z-axis.

#### 2.2.6. FEA Approach

ANSYS Workbench 12 Suite was used on femurs in 15 deg of adduction that were subjected to a 1 kN axial force (i.e., validation versus experiments at a subclinical level and prior published data [17–19]) and a 3 kN axial force (i.e., final analysis at clinical forces) mimicking about 4x body weight for a 75 kg person during the single-leg stance phase of walking. FEA axial stiffness of Cases 1 to 8 was calculated by dividing the axial force applied, i.e. 3 kN, by the axial displacement of the point of force application on the apex of the femur's head. Because the FE model was linear, this was identical to utilizing the slope of the force versus displacement curve. Following initial FEA, Von Mises stress maps for Cases 1 to 9 were evaluated to identify peak stresses. With the “sphere of influence” tool, meshes were refined further around peak stress regions (i.e., several millimeters in all directions), which removed any stress artifacts that sometimes occur at discontinuities in geometry, surfaces that are highly curved, or points of force application.

### 2.3. Experimental Analysis

#### 2.3.1. Femur Characteristics

For all cases, the same single femur was used to provide consistency, as done in prior studies and to represent the effect of surgical changes for a single patient [17–21]. The femur was a large, left, 4th generation artificial femur (Model 3406, Sawbones, Vashon, WA, USA). It had an overall length of 485 mm, a hollow intramedullary canal diameter of 16 mm, a cortical bone with a density of 1.64 g/cm^3^ which was made of e-glass fibers mixed into an epoxy resin, and a cancellous bone (“solid” type) with a density of 0.32 g/cm^3^ which was made of polyurethane. This artificial femur has been previously validated against human cadaveric femurs using a variety of mechanical tests [25–29].

#### 2.3.2. Femur Preparation

The distal condyles of the femur were shaved with a band saw on the medial, lateral, and posterior sides and then potted into a square steel cube filled using anchoring cement used for industrial construction (Flow-stone, King Packaged Materials Company, Burlington, ON, Canada) so that its final working length was 410 mm. This intact femur served as Case 1, which was then mechanically tested. The femur was then altered sequentially to produce the other cases in a particular order and then mechanically tested (Cases 2 to 9) in order to only create screw holes as they became necessary, thereby avoiding empty screw holes which could act as stress risers (Figure 1). As with the FE models, the 12 mm defect (i.e., bone gap) was created using a band saw at 183 mm above the top of the potting cube [1].

#### 2.3.3. Strain Gage Setup

**Figure 2:**
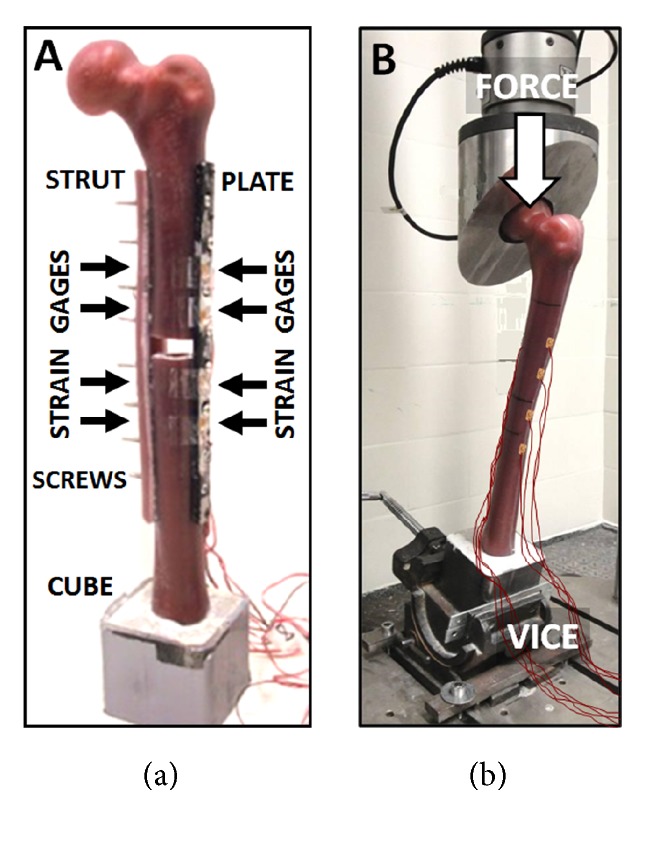
Experimental methods showing (a) a repaired artificial femur for Case 5 with plate, strut, screws, and strain gages and (b) the test setup for applying load via the mechanical tester for Case 1 specimen.

Each femur fixation case was equipped with 350-ohm general purpose linear pattern strain gages (Model CEA-06–125UW-350, Vishay Measurements Group, Raleigh, NC,USA) following the vendor's guidelines (Figure 2(a)). Strain gages were in-line with the long axes of the femur shaft, plate, and strut. Wires were attached to an 8-channel Cronos-PL data acquisition system (IMC Mess-Systeme GmbH, Berlin, Germany). This was connected to a computer for data storage and analysis utilizing FAMOS V5.0 software (IMC Mess-Systeme GmbH, Berlin, Germany). For Case 1, the intact femur had 8 gages mounted, namely, 4 on the lateral side and 4 on the medial side located at 131 mm, 161 mm, 205 mm, and 235 mm from the top of the potting cube. For each subsequent Cases 2 to 9, additional gages had to be fixed on the most lateral and medial exposed surfaces of the femur, plate, and/or strut. This ensured that there were always 4 lateral and 4 medial gages on the specimen at the same vertical locations that matched Case 1.

#### 2.3.4. Mechanical Loading

Each femur fixation case was positioned in 15 deg of adduction in the frontal plane and aligned vertically in the sagittal plane to replicate the single-legged stance phase of gait (Figure 2(b)) [1, 17–22, 29]. Distally, each femur fixation case was fixed firmly in an industrial vice. Proximally, the femur head was placed into a smooth steel cup within which it could rotate freely. An axial (i.e., vertical) force was applied to the top of the femur head utilizing force control (waveform = linear, max force = 1 kN, rate = 100 N/s, preload = 100 N). This force was below what may be physiological for numerous daily activities; however, it kept the specimen within the linear elastic zone and prevented damage. Tests were done 3 times for 90 s each, and an average was taken of the reading from each strain gage for the middle 40 s interval. Tests were performed on an Instron 8874 mechanical tester (Norwood, MA, USA). Similar mechanical loading regimes and strain gage methods were previously used on artificial and human cadaveric femurs and other long bones and implants [1, 17–22, 29–31].

## 3. Results

### 3.1. FEA versus Experimental Validation Using Von Mises Strains

**Figure 3:**
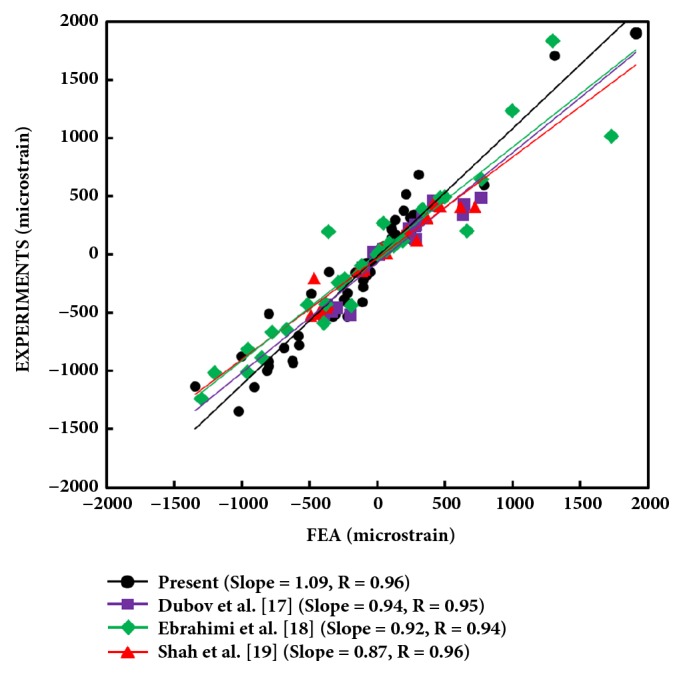
FEA versus experimental strains used for FE model validation at 1 kN and from similar previous femur fracture repair configurations studied by the current senior authors [17–19]. Data from Cases 1 to 9 are combined in a single graph because the same basic FE model was used for each case. Positive and negative values indicate, respectively, tensile and compressive strains. Equivalent elastic (Von Mises) strains are shown. Perfect agreement would be indicated by a slope =1 and a correlation coefficient R = 1.

To validate FEA at 1 kN of axial force with femurs in 15 deg of adduction, all FEA versus experimental Von Mises strains were graphed together for Cases 1 to 9, since the data were from the same basic FE model (Figure 3). The slope = 1.09 and the linear correlation coefficient R = 0.96, both approaching the ideal value of 1; thus, there was strong agreement between FEA and experiments. The graph also showed that present strain data were in excellent agreement compared to previous FEA versus experimental strains from similar femur fracture repair configurations developed by the current senior authors [17–19].

### 3.2. FEA Axial Stiffnesses

**Figure 4:**
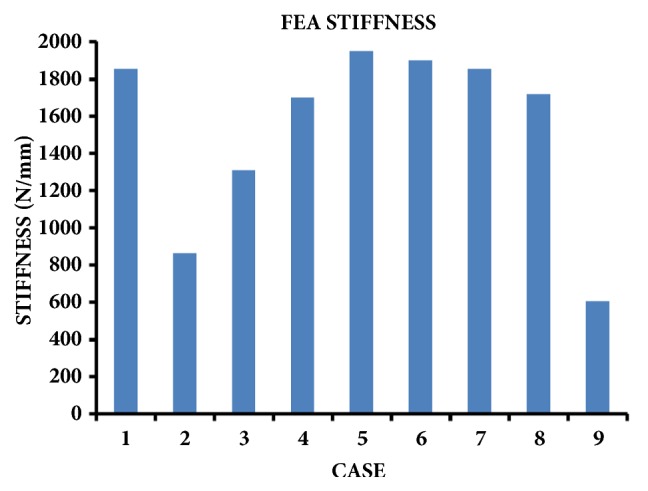
FEA axial stiffnesses at 3 kN of axial force for Cases 1 to 9.

FEA axial stiffnesses are given for 3 kN of axial force applied to femur/plate/strut constructs oriented in 15 deg of adduction (Figure 4). Compared to Case 1 (intact femur) with an axial stiffness of 1853 N/mm, Cases 5 to 7 achieved about the same axial stiffness, whereas Cases 2 and 9 had the lowest axial stiffness. For a full length bone strut there was a substantial influence of the number of screws on axial stiffness (Cases 2 to 5), but when using all 8 screws there was little influence of bone strut length on axial stiffness (Cases 5 to 8).

### 3.3. FEA Von Mises Stresses

**Figure 5:**
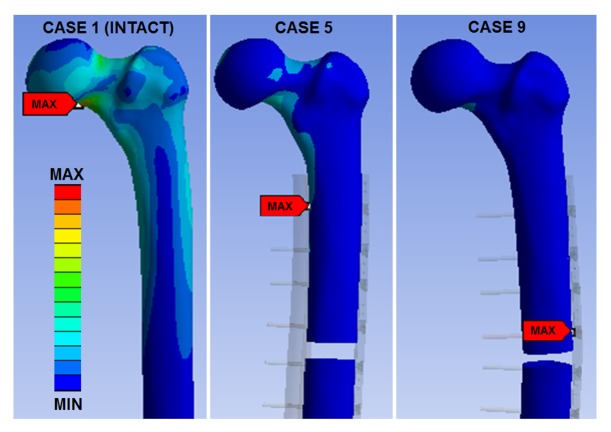
FEA stress maps of the femur cortical bone for an axial force of 3 kN for Case 1 (i.e., intact), Case 5 (i.e., stiffest), and Case 9 (i.e., softest). Equivalent elastic (Von Mises) stresses are shown. Red arrows are peak stresses. Internal stresses are not shown. Stress levels are not shown on the colour scale to avoid figure clutter, but the same colour scale applies to all FE cases.

FEA peak Von Mises stresses and their locations were computed for 3 kN of axial force applied to femur/plate/strut constructs oriented in 15 deg of adduction (Table 1). The concave trend from Cases 2 to 9 in peak stresses of the femur, strut, plate, and screws was opposite to the convex trend in axial stiffnesses (i.e., stress and axial stiffness were inversely related). Consequently, Cases 1, 5, and 9 are important examples (Figure 5). Case 1 (intact femur) had the lowest peak bone stress of 40 MPa versus all other cases (i.e., 42 - 171 MPa), making them all more vulnerable to fracture. Case 5 was the least at risk for fracture, since it had the lowest plate stress, second lowest screw stress, and third lowest strut stress. Case 9 was most at risk for fracture, since it had the highest stresses for the femur, plate, and screws.

## 4. Discussion

### 4.1. Comparison to Prior Studies

Lindsey et al. [13] repaired a 30 mm in vivo canine femur midshaft defect group using a titanium intramedullary nail plus a cancellous-filled cylindrical titanium mesh cage. At 18 weeks postoperatively, this group achieved 73% of the torsional stiffness and 83% of the torsional strength found in contralateral intact femurs, whereas the unfilled defect control group showed no notable bone formation or mechanical stability. This finding agrees with present data, which demonstrated that peak bone and implant stresses that ostensibly increase risk of failure actually occurred in the immediate vicinity of the midshaft defect (i.e., level #4 and #5) in 10 instances for the femur, the strut, and the screws (Table 1). However, their investigation did not assess the influence of a medial bone strut, the location of screws, the number of screws, or a plating method on biomechanical properties, as done currently.

Wieding et al. [16] used an FE model to assess a 30 mm defect in the distal third of a “human-like” artificial femur, mounted an angular-stable osteosynthesis plate on the lateral side fixed with 3 proximal screws in the shaft and 4 distal screws in the condylar region, and filled the defect with a cylindrical hollow titanium scaffold with a 0.5 mm wall thickness; this was closest to the current Case 9, albeit with an unfilled defect. They found that a physiological axial hip force of 1880 N generated 15 times more “collapsing” displacement of the defect itself versus an intact femur's motion at the equivalent location on the bone. In other words, a femur having a defect repaired with a scaffold plus a lateral plate is still much less stiff versus an intact femur, which agrees with the current Case 9 (606 N/mm) being much less stiff than the present intact femur (1853 N/mm) (Figure 4). However, their study did not evaluate medial bone struts, the location of screws, or the number of screws, as done presently.

Talbot et al. [1] assessed a “human-like” artificial femur defect model using 2 repair constructs directly comparable to the present study. They created a 12 mm midshaft defect and repaired it with a 224 mm long 12-hole locking plate mounted laterally with 8 bicortical screws, as well as a 214 mm long 12 hole nonlocking plate mounted laterally with 8 bicortical screws plus a 165 mm long artificial allograft strut mounted medially. Regarding axial stiffness, their intact femur result (1500 N/mm) was somewhat lower than the current Case 1 (1853 N/mm), their plate alone technique (700 N/mm) was most similar to the present configuration of Case 9 (606 N/mm), and their plate plus strut technique (1442 N/mm) was closest in configuration to the current Case 6 (1900 N/mm) (Figure 4). Regarding stresses, their plate alone specimens all failed at the most distal screw hole (5 of 5 cases) which was consistent with current peak stress locations for Case 9 (Figure 5, Table 1). Any differences between their results versus present data are due to the following variations in geometry, material properties and loading conditions: (a) they used the third generation composite femur (compressive E = 7.6 GPa; density = 1.7 g/cm^3^), which has identical geometry but different cortical bone properties than the current fourth generation composite femur (compressive E = 16.7 GPa; density = 1.64 g/cm^3^); (b) their plates were 13% shorter than current ones but had 4 extra screw holes that were empty versus present plates; and (c) they used 18 deg of adduction for axial force, unlike the present 15 deg of adduction.

Moazen and colleagues [32] used FEA on various repair techniques for femurs with a 10 mm midshaft fracture gap, oriented in 10 deg of adduction and subjected to 2300 N of axial force, which is similar to the current study. However, they focused on fractures near the tip of a previously existing primary hip stem which was not within the current study's scope, and they did not assess medial bone struts or the number and distribution of screws as done here. Nevertheless, for repairing a fracture gap below the tip of a previously existing primary hip stem, they found that an 8-hole lateral locking plate combined with an 8-hole anterior locking plate had much greater axial stiffness (2400 N/mm) than either an 8-hole lateral locking plate alone (1100 N/mm) or a 10-hole lateral locking plate alone (1300 N/mm). Moreover, they demonstrated that if a long revision hip stem reaching across the fracture gap was also used as part of surgical repair, axial stiffness was even further increased with or without a lateral locking plate (range, 3500-3700 N/mm). This means that any plating technique that is augmented using circumferential reinforcements around a fracture gap (e.g., additional plates) or longitudinal reinforcements across a fracture gap (e.g., long revision stems) will inevitably minimize in-line interfragmentary motion and, hence, increase axial stiffness, compared to any type of single plate applied alone. This agrees with current data which showed that a lateral metal plate plus a medial bone strut having any length, any number of screws, and any distribution of screws (Cases 2–8) still generated a greater axial stiffness than a lateral metal plate alone even with all its screw holes occupied (Case 9) (Figure 4). They also reported that peak Von Mises stresses (range, 136-1258 MPa) on the plates for all cases were located at the most distal occupied screw hole above the fracture gap, which agrees with present findings that screw holes 1, 2, or 3 above the fracture gap were always the location of peak Von Mises stresses (range, 122 - 1039 MPa) on the plate regardless of the construct configuration (Table 1).

Moazen et al. [33] also performed an FEA study of femur fracture plating in the presence of a prior hip stem by assessing plate material (titanium vs. steel), plate thickness (4.5 vs. 5.6 mm), plate type (nonlocking versus locking), screw configuration (number and distribution), and loading mode (axial and torsional). A hollow cylinder modelled the femur shaft, a proximal solid rod modelled the hip stem, and an oblique line cutting through the cylinder several diameters below the rod modelled the fracture. An axial bending force of 572 N was applied to the top of the rod at 11 deg to simulate femur adduction during walking, whereas a torque of 35 Nm was applied about the central axis to mimic femur torsion. However, they did not model a segmental defect (i.e., fracture gap) or assess medial bone struts. Their plating technique with the most screws (i.e., 9 of 12 screw holes were filled) that also spanned the entire plate length (i.e., the most proximal and distal screw holes were filled) produced over 2x more axial bending and torsional stiffness versus all other repairs regardless of plate material, thickness, or type. Conversely, their plating method with the fewest screws (i.e., 5 of 12 screw holes were filled) that were also most closely spaced together (i.e., 4 screw holes were filled at about the middle third of the plate) generated the lowest axial bending and torsional stiffness. This demonstrated that more screws which are more widely distributed increased mechanical stiffness. This corroborates present data which showed that Case 5 (i.e., 8 of 8 screw holes were filled) had the greatest axial stiffness, whilst Case 2 (i.e., 2 of 8 screw holes were filled) had the lowest axial stiffness, regardless of bone strut length (Figure 4). They also reported for their highest stiffness repair method that peak Von Mises stresses on the plate occurred around screw holes at the same level or just below the fracture, which disagrees with the present highest stiffness construct (Case 5) which had peak Von Mises stresses on the plate just above the fracture gap at screw hole 3 (Table 1); this difference may be due to current buttressing by the medial bone strut that the prior authors did not model.

Moazen and coworkers [34] also did a comparison of the relatively new technology of “far cortical locking” (FCL) plates versus traditional locking plates for repairing unstable fracture gaps at the femur midshaft in the presence of a prior hip stem. FCL technology uses bicortical screws with reduced shaft diameter to permit micromotion at the near cortex between the screw shaft and bone pilot hole (but full engagement with bone at the far cortex), which can increase in-line interfragmentary motion at the fracture gap to the recommended range of 0.2-1 mm to enhance callus formation for secondary-type healing. Although the current authors did not evaluate FCL plates, this prior work could provide clues about how this new plating system could further optimize the presently examined surgical methods and results. They experimentally applied up to 700 N of axial force to synthetic femurs that were osteotomized with a 10 mm unstable midshaft fracture gap, oriented in 10 deg of adduction to mimic single leg stance during walking, and repaired using either FCL plates on the lateral shaft (i.e., 6 traditional locking screws above the fracture gap plus 4 FCL screws below the fracture gap) or traditional locking plates on the lateral shaft (i.e., 6 traditional locking screws above the fracture gap plus 4 traditional locking screws below the fracture gap). Their FCL constructs had statistically higher in-line interfragmentary motion on the medial side of the fracture gap as well as lower overall axial stiffness (1.25 mm, 331 N/mm) versus their traditional locking plate constructs (0.8 mm, 443 N/mm), which are substantial differences, respectively, of 56% and 25%. This suggests that FCL could potentially be combined with the current repair methods to further optimize medial bone strut length, as well as the number and the location of FCL and non-FCL screws. This would help researchers and clinicians to design femur fracture repair constructs that generate in-line interfragmentary motion within the ideal range of 0.2-1 mm at the fracture gap, which would promote earlier callus formation, secondary-type healing, patient recovery, and a return to routine activities.

### 4.2. Clinical Implications

The traditional clinical approach has been to choose long bone repair constructs that are as rigid as possible, i.e., “high stiffness” criterion [1, 17–19]. As such, Case 5 would be the most desirable since it provided the greatest mechanical stiffness using a bone strut equal in length to the metal plate in which both are affixed with the maximum number of possible screws (i.e., 8 screws for the present 8-hole plate) compared to Case 1 intact femur (Figure 4). Nonetheless, a reduction in the number of screws to 6 (Case 4) or even 4 (Case 3) still preserved, respectively, 98% and 78% of Case 1 stiffness. This suggests that only a few screws are needed at the extreme ends of the strut for stability, rather than near the segmental defect. Thus, maximal screw hole use may be surgically unnecessary, thereby minimizing the bone loss, stress risers, and stress fractures associated with screw insertion [1, 17–19]. Moreover, the shortest possible bone strut (Case 8) still retained 84% of Case 1 stiffness. This suggests that the mere presence of any medial buttress at the segmental defect site is sufficient for stability. Thus, maximal strut length may be surgically unnecessary, thereby minimizing soft tissue stripping to accommodate a strut, as well as reducing operating time, blood loss, patient discomfort, muscle elevation, and/or bone devascularisation [4].

The more modern clinical approach is to choose long bone repair constructs which are more flexible, i.e., “low stiffness” criterion. This accomplishes two things: it can allow some in-line interfragmentary motion of 0.2-1 mm which is known to enhance callus formation for secondary-type fracture healing, as well as reduce the negative effects of “stress shielding” [34–36]. For example, Cases 2 and 9 had axial stiffnesses (862 and 606 N/mm) that correspond to femoral head displacements of 0.23 and 0.33 mm if only a 200 N toe-touch force was applied, as often recommended to patients immediately after surgery [36]. If these displacements are also similar at the fracture site, they fall within the optimal range for in-line interfragmentary motion (i.e., 0.2 - 1 mm) that is known to enhance callus formation for secondary-type fracture healing [34, 36]. Furthermore, Cases 5 and 7 had stiffnesses that were only 5.1% and 2.5% higher than the intact femur of Case 1 (Figure 4), but the corresponding plate and screw peak stresses were 1.33 - 4.29 times higher than the femur peak stresses (Table 1); thus, this could cause stress shielding of bone, leading to bone atrophy, bone resorption, and implant loosening [35]. Consequently, investigators have argued for the development of devices that closely replicated the properties of the intact host bone or joint. To this end, implants have been fabricated completely from polymer-based composites [35, 37, 38], metal implants have been augmented with low-stiffness material parts [39], or metal fracture fixation implants have been chosen which duplicate the host structure's stiffness [40]. Given current data, researchers may wish to examine a stiffness-matching approach to design implants to address the problem of femur shaft fractures.

Several bone-implant configurations at present may be at risk of sudden catastrophic failure based on how close their bone or implant stresses (Table 1) were to the material's ultimate tensile strength (UTS) or ultimate compressive strength (UCS). Although Von Mises stress is a distortion-based measure, it is a reasonably reliable outcome for comparison to UTS or UCS, as done previously [17–20]. For the femur, Cases 1 to 8 had peak femur stresses of 40 to 113 MPa and, hence, were generally below the UTS of 106 MPa [23] and definitely below the UCS of 157 MPa [23] for artificial cortical bone material, thus minimizing the risk of failure. The exception was Case 9 in which femur peak stress reached 171 MPa just proximal to the defect, thus indicating that some microcracking may have begun to occur. For the cortical bone strut, Cases 2, 3, and 8 may be at risk of failure since their respective strut peak stresses (318, 124, and 127 MPa) substantially exceeded either cortical bone UTS or UCS. For metallic implants, Case 2 at the most distal screw (1033 MPa) and Case 9 at the screw (1680 MPa) and plate (1039 MPa) near the midshaft defect exceeded the UTS of stainless steel (560 MPa) [18].

### 4.3. Addressing Possible Limitations

The potential drawbacks below are typical of in vitro biomechanical studies, which may change the absolute stiffnesses and stresses currently obtained, but which would unlikely change the relative performance between the 9 cases examined.

An artificial femur was used experimentally because of its standardized geometric and material properties [23, 31], and since it agrees with cadaveric bone properties, like overall axial to torsional rigidity ratio (2.11 ± 0.02 versus 2.97 ± 0.71 rad/m^2^), cortical elastic compressive modulus (16.7 versus 11.5-17.0 GPa), cancellous elastic compressive modulus (0.155 versus 0.01-2 GPa), cortical ultimate compressive strength (157 versus 133-193 MPa), and cancellous ultimate compressive strength (6 versus 0.1-100 MPa) [1, 17–23, 26, 27, 41–45]. However, structural variation of cancellous bone along lines of applied stress does not occur in an isotropic artificial femur, as it does in a human femur [46].

The FE model of the artificial femur was linear, isotropic, and homogeneous, which may not fully represent all aspects of a human femur, which is nonlinear, anisotropic, and heterogeneous, as well as potentially osteoporotic [17–21].

Only quasi-static axial force was computationally modelled and experimentally applied; however, the femur, lateral metal plate, and medial bone strut can all experience cyclic forces for many activities of daily living [47].

A “single specimen” strategy was used as done in prior biomechanical studies, since the relative differences between cases are of interest which could represent, say, the femur of a specific patient [17–21, 35]. Moreover, very similar FE models of femur fracture fixation have been validated against experimental strain in several other studies by some of the current senior authors [17–19]. Thus, a single femur that was sequentially altered to create different fracture fixation cases was sufficient as a basic double-check of the validity of the FE model.

Muscle forces were not accounted for computationally or experimentally, although their inclusion would certainly mimic the in vivo situation more accurately; however, the femur mainly experiences axial compression [1, 17–22, 42, 43, 45].

Force-to-failure was not examined, which may be important because elderly and younger people both can undergo femur fractures during the 6.5 million traffic accidents that happen in the USA alone [48].

Relative micromotion of femur fragments at the defect site was not computed or measured, but this may be important in fracture union, since investigators have demonstrated that micromotion of 0.2-1 mm perpendicular to the fracture gap can enhance secondary healing via callus formation and reduce healing time [36].

The hip contact force vector applied at the femoral head computationally and experimentally reduced hip loading essentially to an in-plane 2D phenomenon for the single leg stance phase of gait (i.e., at about 30% of the gait cycle), so that it was composed of one force component acting in the inferior direction whereas the other acted in the lateral direction relative to the femoral shaft's long axis. In reality, the hip contact fore vector also has a force component acting out-of-plane in the posterior direction whose magnitude (6-7% of the 3D resultant hip contact force vector F_R_) is much smaller than the inferior (97-98% of F_R_) and lateral (19-26% of F_R_) force components [49–51]. As such, the 2D simplification has been commonly done previously [1, 17–22, 29, 35, 40, 41, 45], since the proximal femur is mainly under a state of compression during normal walking [52, 53]. However, basic beam theory suggests current data would be slightly modified if the posterior force component was included. Firstly, the posterior force component would have caused the femoral neck and shaft to bend backward a small amount (like a cantilevered beam) to generate higher tensile stresses on anterior surfaces and higher compressive stresses on posterior surfaces of the bone, bone strut, and metal implants. Secondly, the posterior force component would have also created some torque around the femoral shaft's long axis (via the lever arm of the femoral neck) to generate shear stresses in the femoral shaft, bone strut, and metal implants. This would have only marginally altered the overall axial stiffnesses and the Von Mises stresses, while the relative biomechanical performance of the 9 cases examined would probably have remained the same.

## 5. Conclusions

This study computationally and experimentally examined plate and strut fixation of a large femoral midshaft segmental defect. Case 5 consisting of plate and strut fixed with the maximum possible number of screws had the highest axial stiffness and was the least at risk for fracture, since it had the lowest plate stress, second lowest screw stress, and third lowest strut stress. Conversely, Cases 2 and 9 had the lowest axial stiffnesses, which could potentially permit some interfragmentary motion for enhanced fracture healing.

## Figures and Tables

**Table 1:** FEA Von Mises peak surface stresses for a 3 kN axial force for all components of Cases 1 to 9. The location of each peak stress occurred at a particular screw or screw hole “level”.

**Case**	**Peak Von Mises Stress (MPa) at 3 kN of Axial Force**			
	**Femur**	**Strut**	**Plate**	**Screws**
1	40 (inferior neck)	---	---	---

2	113 (level #8)	318 (level #8)	378 (level #2)	1033 (level #8)

3	96 (level #1)	124 (level #7)	325 (level #1)	454 (level #7)

4	42 (level #1)	89 (level #6)	257 (level #1)	293 (level #6)

5	92 (level #1)	75 (level #5)	122 (level #3)	256 (level #4)

6	66 (level #2)	70 (level #7)	283 (level #3)	227 (level #4)

7	86 (level #3)	18 (level #4)	313 (level #3)	288 (level #4)

8	112 (level #4)	127 (level #5)	341 (level #3)	388 (level #4)

9	171 (level #4)	---	1039 (level #3)	1680 (level #4)

## Data Availability

The data used to support the findings of this study are available from the corresponding author upon request.
